# Pharmacokinetics of Dolutegravir, nucleoside analogues, and intracellular metabolites using plasma separation cards: A comparative analysis with traditional sampling methods in healthy volunteers

**DOI:** 10.1371/journal.pone.0341252

**Published:** 2026-01-23

**Authors:** Beth Thompson, Laura J. Else, Laura Dickinson, Stacey Edick, Ashley Zyhowski, Ken Ho, Leslie Meyn, Sujan Dilly-Penchala, Victoria Shaw, Ian McGowan, Saye Khoo, Rhonda M. Brand

**Affiliations:** 1 Centre for Experimental Therapeutics, University of Liverpool, Liverpool, United Kingdom; 2 University of Pittsburgh School of Medicine, Pittsburgh, Philadelphia, United States of America; 3 Magee-Womens Research Institute, Pittsburgh, Philadelphia, United States of America; 4 Synklino A/S, Copenhagen, Denmark; Rush University, UNITED STATES OF AMERICA

## Abstract

**Objectives:**

Adherence to antiretroviral therapy is essential for achieving viral suppression. Plasma separation cards (HemaSep; HS-DBS) provide advantages for pharmacokinetic (PK) analysis and adherence monitoring, including simplified sample collection. This study compared the PK of dolutegravir (DTG), nucleoside reverse transcriptase inhibitors (NRTIs), and their intracellular metabolites in the dried plasma and cellular fractions of HS-DBS against the appropriate gold-standard matrices: liquid plasma for parent drugs and Whatman DBS (WM-DBS) for NRTI metabolites.

**Methods:**

The APT-POCT-01 clinical trial (NCT04302896) is an open-label study assessing drug concentrations following cessation in healthy volunteers. Participants were randomized (1:1) to receive DTG/FTC/TAF or DTG/3TC/TDF for 15 days. Paired liquid plasma (L-pL), HS-DBS, and WM-DBS samples were collected on Day 15 and following treatment cessation (0–336 hours post-final dose).

**Results:**

29 individuals were included in the PK analysis (15-TDF/14-TAF). Tenofovir diphosphate (TFV-DP) was quantifiable up to 14 days post-cessation in HS-DBS (TAF/TDF) and WM-DBS (TDF) (HS-DBS t½ > 17 days, WM-DBS t½ = 15 days). 3TC-TP and FTC-TP were eliminated more rapidly. Nucleoside di/triphosphate concentrations were 3–7-fold higher, with prolonged half-lives (TFV-DP, FTC-TP), compared with WM-DBS. TFV-DP levels were ~12-fold higher with TDF compared to TAF. For NRTI and DTG, HS-plasma resulted in 1.8-fold higher exposures compared to L-pL. Measurable HS-DBS concentrations were correlated with L-pL and WM-DBS, with Bland-Altman analysis indicating agreement between methods.

**Conclusions:**

This study provides important insights into the elimination kinetics of NRTI, their intracellular metabolites and DTG. Plasma separation cards are a promising alternative for adherence monitoring, enabling simultaneous quantification of parent and intracellular moieties from a single sample. Differences in TFV-DP levels between TDF and TAF regimens, and DBS sampling method, underscore the need for matrix and regimen-specific interpretation to validate adherence benchmarks.

## Introduction

Adherence is a known predictor of the effectiveness oral ART and success of oral daily preexposure prophylaxis (PrEP). Quantification of antiretrovirals in individuals receiving ART or PrEP can be used as an objective measure of adherence.

Nucleoside/nucleotide reverse transcriptase inhibitors (NRTI), including tenofovir (TFV), emtricitabine (FTC) and lamivudine (3TC), are eliminated rapidly from plasma with terminal elimination half-lives between 10–15 hours [1]; such that, objective drug measurements following random or opportunistic sampling do not easily distinguish between recent dose ingestion and sustained adherence over a prolonged time period.

Dried blood spots (DBS) have been used as metrics for NRTI adherence. Tenofovir-diphosphate (TFV-DP) has a half-life in DBS of approximately 17 days, which translates to a predicted 25-fold accumulation from first dose to steady state with standard daily dosing of TDF [1,2]. The accumulation of TFV-DP in DBS over a prolonged period has enabled investigators to establish “concentration gradients” that distinguish different degrees of adherence, such as continuous daily dosing (7 doses/week), sub-optimal dosing (2–3 doses/week) and potential non-adherence (<2 doses/week) [1]. TFV-DP levels in DBS (Whatman Protein Saver cards) have proven to be a significant predictor of PrEP effectiveness in MSM populations in studies such as iPrEx OLE, ATN 110/113, and PrEP-Demo [3–5].

Emtricitabine-triphosphate (FTC-TP) has a relatively short half-life of approximately 1.5 days in DBS and may serve as a marker of recent intake [2,6] . As TFV and FTC are often co-administered as part of fixed dose combinations, they can provide both cumulative and recent dosing information. For instance, white coat dosing may be suspected if FTC-TP is quantifiable, but TFV-DP is low or unquantifiable. Currently there are no data detailing the kinetics of lamivudine triphosphate (3TC-TP) in DBS.

HemaSep™ filter cards (Ahlstrom-Munksjö, Helsinki, Finland) are a novel DBS technology that can separate plasma from whole blood upon contact within minutes without need for centrifugation. This permits simultaneous quantification of both the parent NRTI (in plasma) and its phosphorylated metabolite (nucleoside di-/triphosphate; cellular fraction) from the same sample to derive recent and cumulative adherence metrics.

Here we report the pharmacokinetics (PK) of dolutegravir (DTG), nucleoside analogues (TFV, FTC, 3TC) and their nucleoside di-/triphosphates in the cellular components of dried blood and plasma using plasma separation cards (HemaSep™). Drug and phosphorylated metabolite concentrations obtained using this novel sampling technology were compared to those derived from established sampling approaches, including liquid plasma (for parent drugs) and Whatman DBS (for nucleoside di/triphosphates). Concentrations were measured over 14 days post- treatment cessation in a cohort of healthy volunteers (APT-POCT-01) receiving commonly prescribed NRTI-based regimens.

## Materials and methods

### Ethics

The study was conducted in accordance with the Declaration of Helsinki and applicable local and US regulatory requirements. Participants were asked to provide written informed consent, and the study protocol and consent documents were approved by the University of Pittsburgh Institutional Review Board (CR19100009−005).

### Study design

APT-POCT-01 (ClinicalTrials.gov identifier - NCT04302896) is an open-label study performed in a healthy volunteer cohort which evaluated the decay PK of the nucleoside analogues (TFV, FTC, 3TC) and their phosphorylated metabolites in various matrices, including plasma, urine, saliva and DBS (HemaSep, Whatman) over a period of fourteen days following drug intake cessation [7]. The full trial protocol and the Statistical Analysis Plan can be accessed in the Supplementary Material. This article and supplementary material are open access.

Study participants were recruited at the Magee-Womens Hospital Clinical Translational Research Centre (MWH CTRC) between 31^st^ August 2020 and 27^th^ September 2021. Individuals ≥18 years of age were eligible if they were HIV-1 seronegative at screening, were in general good health and, if female, were willing to use an effective method of contraception (IUD, hormonal contraceptives) throughout the study duration. Participants that were pregnant, breastfeeding, or had been using PrEP within the last 3 months were excluded.

### Study procedures

**Fig 1:**
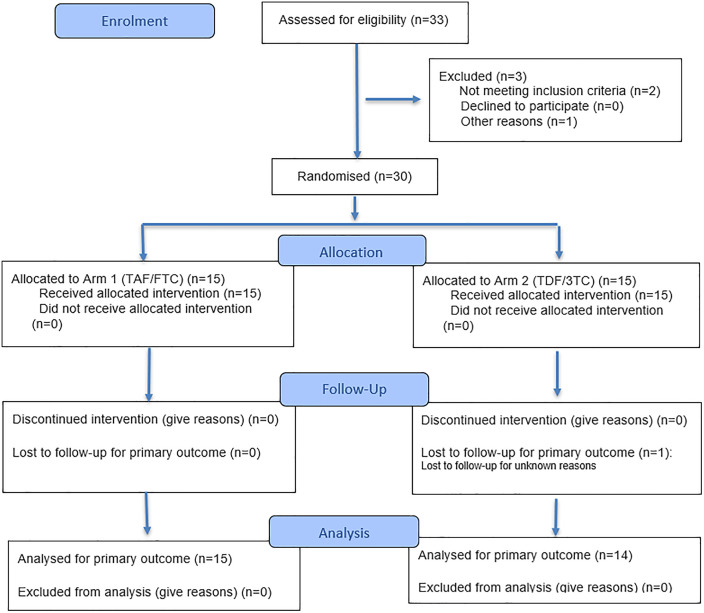
CONSORT 2025 flow diagram of the progress through the phases of a randomised trial of two groups (that is, enrolment, intervention allocation, follow-up, and data analysis) [8].

After screening, thirty participants were randomised (1:1) into one of two treatment arms. Arm 1 participants received dolutegravir 50 mg (Tivicay®; ViiV Healthcare BV) in combination with emtricitabine 200 mg/tenofovir alafenamide 25 mg (Descovy®; Gilead Sciences Ltd) once daily for 15 days. Arm 2 received dolutegravir 50 mg in combination with tenofovir disoproxil fumarate 300 mg (Viread®; Gilead Sciences Ltd) and lamivudine 300 mg (Apotex, FL, USA) once daily for 15 days (see Fig 1). Four of 15 doses (dose 1, 2, 8 and 15) were directly observed in clinic. All other doses were self-administered and recorded by the participant, who maintained a dosing diary to monitor adherence.

Paired liquid plasma and DBS samples were collected during the “dosing phase” (day 1, 2 and 8), and after ingestion of the final dose on days 15 (samples taken at 0, 1, 4 hours post-dose), 16, 17, 18, 19, 22 (equivalent to 24, 48, 72, 96, 168, 336 hours post-dose, respectively) during the “drug cessation phase”. Specimens were collected and processed at MWH CTRC. Whole blood was collected in 10mL K_2_EDTA vacutainer tubes, inverted several times, and spotted onto two types of filter card – HemaSep card (Ahlstrom-Munksjö, CytoSep®HV 1668) and Whatman 903 Protein Saver Card (Whatman; Fisher Scientific).

### Sample processing

For Whatman cards (WM-DBS), exactly 50 µL of whole blood was spotted onto the centre each designated circle (5 spots per card) and air dried in a card holder at room temperature for at least 2 hours. Similarly, exactly 100 μL of whole blood was spotted onto the centre of each inner (hatched) circle of the HemaSep (HS-DBS) card in a single fluid motion (2 spots per card) and the red blood cell (RBC) and plasma components were allowed to separate. Complete separation of plasma is achieved within 2 minutes. The HS-DBS cards were then left to air dry on the drying rack for at least 2 hours. Once fully air dried the DBS cards were placed in individual sealed bags containing humidity indicators and desiccant.

Plasma was obtained from the same K_2_EDTA vacutainer tubes as the DBS. After spotting, the vacutainer tube was centrifuged at 1500 x g for 10 minutes at 4°C and the plasma aliquoted. All samples were stored at −70°C pending analysis.

### Drug quantification (Bioanalysis)

All analytes were measured using validated LC-MS assays [9]. Nucleoside di-/triphosphates were extracted and quantified from the inner cellular fraction of the HS-DBS (inner spot; HS-C) as previously described, using a combination of protein precipitation (70:30 methanol:20% formic acid in water) with a solid phase extraction (SPE) clean-up step [10]. Nucleoside di-/triphosphates were also quantified from WM-DBS, serving as the gold standard DBS matrix (1 x 6 mm sub-punch) using an adaptation of this methodology. The calibration ranged between 1.25–250 pmol/sample, and concentrations were expressed as fmol/punch. The calibration range was later extended to 0.25–250 pmol/sample in order to capture lower concentrations of TFV-DP from TAF samples.

Parent NRTI (TFV, FTC, 3TC) were quantified from the outer plasma ring of HS-DBS (HS-pL) and from paired liquid plasma samples (L-pL), the latter serving as the gold standard metric. The NRTI were extracted from L-pL as previously described [11,12]. For HS-pL, the entire outer ring was excised and extracted with 80:20 (v/v) acetonitrile:0.1% formic acid, after which samples were dried and reconstituted in 300 µL 99:1 (v/v) H_2_O:ACN. The calibration curves were 1–1000 ng/mL (TFV) and 5–5000 ng/mL (FTC/3TC) in L-pL; and 0.05–50 ng/sample (TFV), 0.25–250 ng/sample (FTC/3TC) for HS-pL, respectively.

Quantification of DTG from L-pL was achieved using a liquid-liquid extraction method (TBME), as previously described [13]. For HS-pL, a pre-soaking step (500 µL 1.8 mg/mL EDTA in H_2_O) was incorporated prior to the addition of TBME, after which extracts were dried down and reconstituted in 500 µL 80:20 (v/v) MeOH:0.1% formic acid. DTG calibration curves were 10–10000 ng/mL (L-pL) and 0.5–500 ng/sample (HS-pL).

To compare with L-pL, measured HS-pL concentrations were normalised to ng/mL by assuming an approximate outer ring plasma volume of 50 µL. This was based on a haematocrit level of ~50% which is consistent with normal physiological ranges for healthy adults [14].

### Pharmacokinetic analysis

Pre-dose samples taken at baseline (Day 1) that were below the assay lower limit of quantification (LLQ) were set as 0 ng/mL. Post-dose samples that were <LLQ were assigned half LLQ values, and for consecutive post-dose samples <LLQ, the first was assigned a half LLQ value and any subsequent samples excluded from data analysis. PK parameters including half-life (t½), C_max_, T_max_, and area under the concentration time curve to the last measurable time point (AUC_last_) were calculated using non-compartmental methods (Phoenix 64 WinNonlin, version 8.3).

### Statistical analysis

Quantifiable concentrations were log transformed, and a coefficient of determination (r^2^) used to determine the presence of statistically significant relationships in drug exposures between matrices. Geometric mean ratios (GMR) were calculated to assess differences between the PK parameters derived from paired HS-pL and L-pL (DTG/3TC/FTC/TFV) and paired HS-DBS and WM-DBS (3TC-TP/FTC-TP/TFV-DP). GMR were calculated with WM-DBS and L-pL serving as the reference matrix. Confidence intervals (CIs) were derived from the logarithms of individual ratios, and the resulting limits were subsequently back-transformed to provide linear values. Differences in PK parameters were considered statistically significant when the 95% CI did not cross the value of 1.

The agreement between the two methods was evaluated using Bland-Altman plots (SigmaPlot version 14.5, Systat Software, Inc). Bland–Altman statistics, including the mean difference and limits of agreement, were calculated on log-transformed concentrations and then back-transformed to yield fold-change estimates.

## Results

### Study population

Demographics and clinical measurements of the study population are summarised in Table 1. A total of 29 patient PK profiles were evaluated (15 subjects in Arm 1 and 14 subjects in Arm 2). Among the participants, 21 (72%) were female at birth and 26 (90%) were Caucasian. The median (range) age was 36 years (20–67), and weight was 73.0 kg (49.0–97.5). There were no substantial differences in mean clinical measures between the 2 arms.

**Table 1:** Participant demographics and clinical characteristics (n = 29).

Parameter	Arm 1 (TAF|FTC)	Arm 2 (TDF|3TC)	Total	P value^b^
N (%)	15 (51.7)	14 (48.3)	29 (100)	
Age (yr)	34 (21-58)	38 (20-67)	36 (20-67)	0.948
Weight (kg)	77.1 (56.2-97.5)	68.0 (49.0-97.5)	73.0 (49.0-97.5)	0.407
Height (cm)	165.1 (152.4-185.4)	165.1 (142.2-185.4)	165.1 (142.2-185.4)	0.878
BMI (kg/m^2^)	29.5 (20.2-36.5)	25.9 (20.4-29.9)	26.7 (20.2-36.5)	0.138
Sex at birth, N (%)				
Female	13 (86.7)^a^	8 (57.1)	21 (72.4)	0.075
Male	2 (13.3)	6 (42.9)	8 (27.6)	
Ethnicity				
White	12 (80.0)	13 (92.9)	25 (86.2)	0.316
Black African-American	1 (6.7)	0 (0.0)	1 (3.4)	
Asian	2 (13.3)	1 (7.1)	3 (10.3)	
Creatinine (mg/dL)	0.76 (0.59-1.10)	0.87 (0.69-1.11)	0.80 (0.59-1.11)	0.029
Bilirubin (mg/dL)	0.50 (0.30-0.70)	0.55 (0.30-1.60)	0.50 (0.30-1.60)	0.168
ALT (U/L)	13.0 (9.0-49.0)	13.5 (8.0-34.0	13.0 (8.0-49.0)	0.599
AST (U/L)	15.0 (10.0-24.0)	17.5 (10.0-21.0)	17.0 (10.0-24.0)	0.417

**Fig 2:**
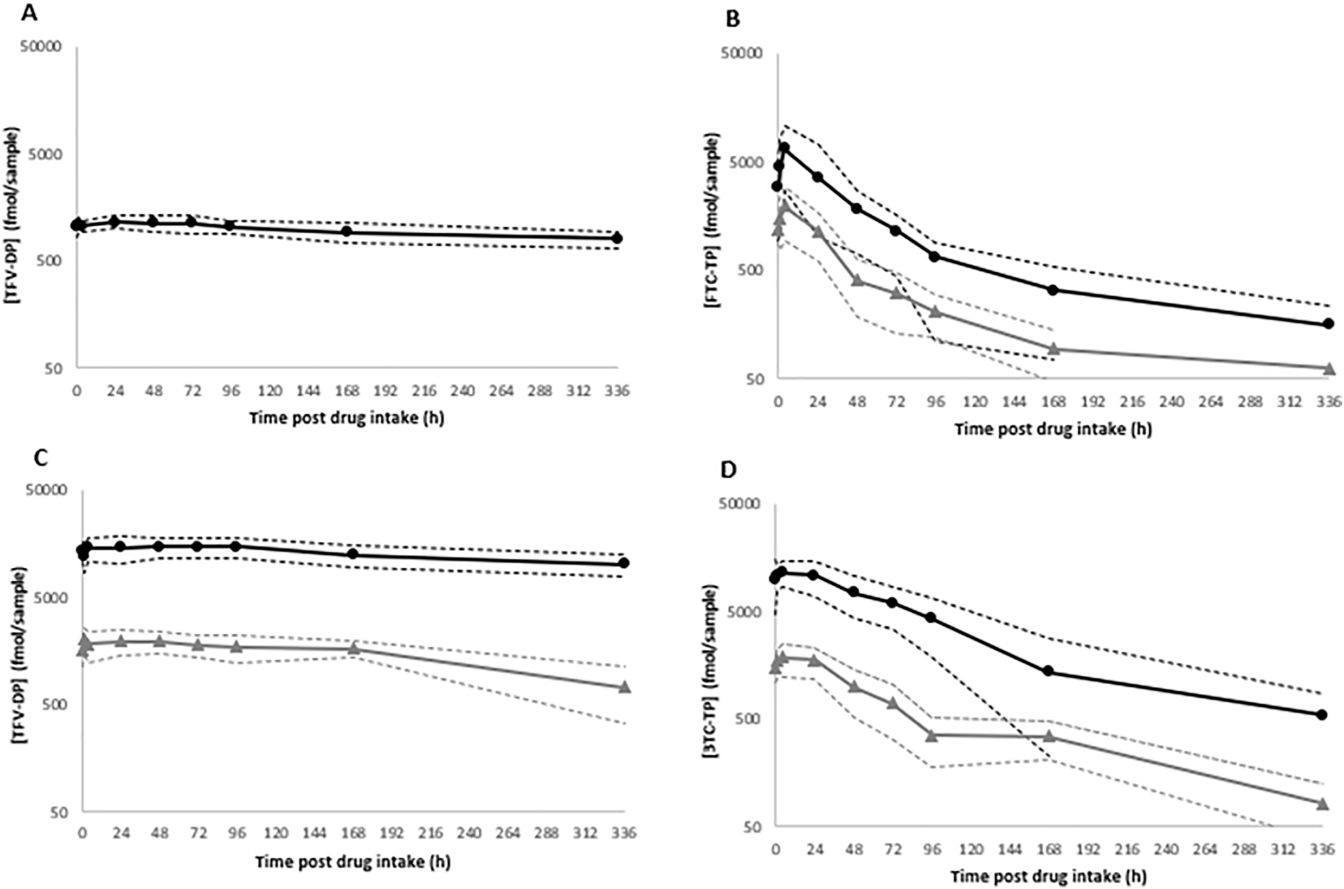
Geometric mean (95% confidence interval) nucleoside di-/triphosphate concentrations in HS-C and WM-DBS samples for (A) TFV-DP_TAF_, (B) FTC-TP, (C) TFV-DP_TDF_ and (D) 3TC-TP during the 14-day drug intake cessation period. HS-C is indicated with a solid black line (circles) and WM with a grey line (triangles). Metabolite concentrations are presented in fmol/sample (HS-C = 12 mm punch; WM-DBS = 6 mm punch). Detectable concentrations below the assay limit of quantification (HS-C = 250 fmol/sample, WM-DBS = 62.5 fmol/sample) are expressed as half LLQ values. Fig 2A displays HS-C data only, as all WM-DBS had undetectable TFV-DP_TAF_.

**Fig 3:**
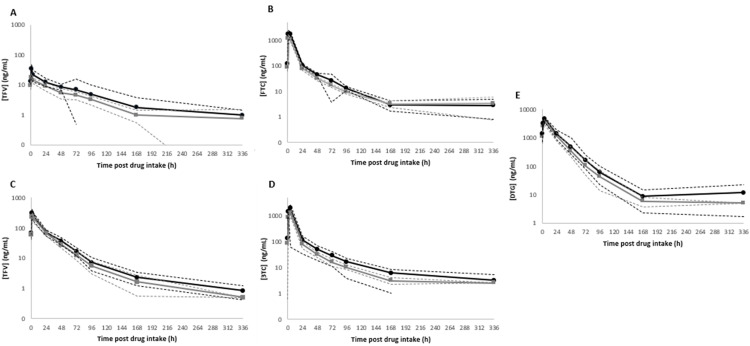
Geometric mean (95% confidence interval) drug concentrations in dried (HS-pL) and liquid plasma (L-pL) for (A) TFV_TAF_, (B) FTC, (C) TFV_TDF_ (D) 3TC and (E) DTG during the 14-day drug intake cessation period. HS-pL is indicated with a solid black line (circles) and L-pL with a grey line (squares). Drug concentrations are presented in ng/mL. Concentrations below the assay limit of quantification for HS-pL (TFV – 0.05 ng/50 µL, TAF – 0.025 ng/50 µL, FTC/3TC – 0.25 ng/50 µL) and L-pL (TFV – 1.01ng/mL, TAF – 0.5ng/mL, FTC/3TC – 5ng/mL) are expressed as half LLQ values.

**Fig 4:**
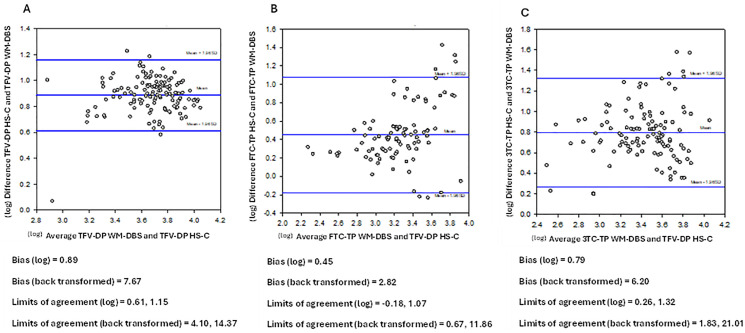
Bland Altman plots comparing WM-DBS and HS-C for TFV-DP_TDF_ (A), FTC-TP (B) and 3TC-TP (C) as a measure of agreement between the two methods. Concentration data were log-transformed, and the mean differences and limits of agreement are presented as back-transformed ratios.

**Fig 5:**
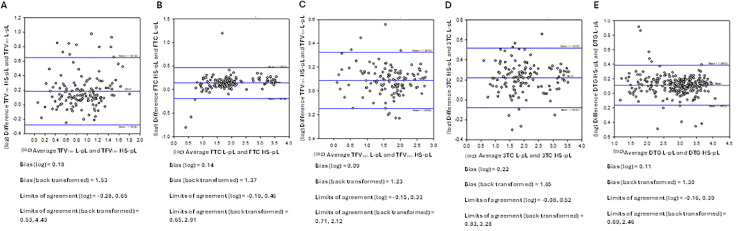
Bland Altman plots comparing L-pL and HS-pL for TFV_TAF_ (A), FTC (B), TFV_TDF_ (C), 3TC (D) and DTG (E) as a measure of agreement between the two methods. Concentration data were log-transformed, and the mean differences and limits of agreement are presented as back-transformed ratios.

Geometric mean (GM) 95% confidence interval (95% CI) concentrations of the nucleoside di-/triphosphates in DBS (HS-C and WM-DBS) over the 14-day cessation period are shown in Fig 2 (individual profiles are shown in S3 Fig). NRTI drug concentration profiles in HS-pL and L-pL are depicted in Fig 3 (individual profiles are shown in S4 Fig). The PK parameters for the nucleoside di-/triphosphates and parent drugs are summarised in Table 2 and Table 3, and Bland Altman plots are depicted in Fig 4 (nucleoside di-/triphosphates) and Fig 5 (NRTI and DTG), respectively. Correlations were created using log-transformed data (see S1 and S2 Figs). Individual participant profiles can be found in S3 and S4 Figs.

**Table 2:** Geometric mean (95% CI)[%CV] pharmacokinetic parameters for TFV-DP_TAF_, FTC-TP (Arm 1) and TFV-DP_TDF_, 3TC-TP (Arm 2) in HS-C and WM-DBS over 14 days drug intake cessation in healthy volunteers.

Arm 1	TFV-DP_TAF_			FTC-TP		
	HS-C	WM-DBS	GMR (95% CI)	HS-C	WM-DBS	GMR (95% CI)
**C**_**max**_ (fmol/punch)	1374 (1222,1526) [21.4]	<LLQ	–	7460 (2850, 12070) [90.4]	1900 (949.83, 2851) [81.5]	**3.92** (2.25, 6.84)
**T**_**max**_ (h)	14.72 (−3.39, 32.83) [105.6]	–	–	3.03 (2.40, 3.66) [36.5]	5.06 (−4.12, 14.25) [193.1]	0.59 (0.31, 1.11)
**C**_**24**_ (fmol/punch)	1155 (1003, 1306) [25.2]	<LLQ	–	3562 (−70.35, 7194) [136.6]	1141 (594.06, 1688) [65.9]	**3.19** (1.63, 6.24)
**C**_**48**_ (fmol/punch)	843.13 (595.43, 1090.83) [47.1]	<LLQ	–	1838 (993, 2684) [75.6]	405.42 (186.24, 624.60) [63.7]	**4.86** (2.59, 9.12)
**C**_**72**_ (fmol/punch)	1122 (906.59, 1337) [33.4]	<LLQ	–	1153 (701.82, 1605) [68.3]	305.51 (131.45, 479.57) [66.8]	**4.27** (2.26, 8.06)
**C**_**96**_ (fmol/punch)	1039.12 (909.82, 1168) [22.3]	<LLQ	–	658.73 (434.29, 883.17) [59.2]	208.23 (119.28, 297.19) [42.6]	**3.16** (1.46, 6.85)
**AUC**_**last**_ (fmol.h/punch)	245831 (177654, 314007) [45.1]	<LLQ	–	334894 (178313, 491474) [75.8]	71373 (46299, 96448) [58.2]	**4.69** (3.03, 7.26)
**T**_**last**_ (h)	259.21 (207.93, 310.50) [34.0]	–	–	279.30 (240.38, 318.21) [26.4]	89.86 (40.44, 139.27) [84.6]	**3.11** (2.16, 4.48)
**t½** (h)	424.93 (280.27, 569.58) [44.4]	–	–	76.34 (34.46, 118.23) [85.2]	21.91 (−2.65, 46.46) [115.1]	**4.42** (2.42, 8.07)
**Arm 2**	**TFV-DP**_**TDF**_			**3TC-TP**		
	**HS-C**	**WM-DBS**	**GMR (95% CI)**	**HS-C**	**WM-DBS**	**GMR (95% CI)**
**C**_**max**_ (fmol/punch)	17166 (13829, 20502) [34.6]	2271 (1716, 2827) [42.4]	**7.56** (6.68, 8.54)	14354 (9037, 19671) [62.6]	2089 (1485, 2692) [47.7]	**6.83** (4.69, 9.93)
**T**_**max**_ (h)	27.30 (7.82, 46.78) [88.5]	18.20 (−0.21, 36.61) [84.4]	1.20 (0.40, 3.60)	9.47 (−3.66, 22.61) (160.0]	4.89 (−0.06, 9.83) [111.6]	2.17 (0.79, 5.97)
**C**_**24**_ (fmol/punch)	14293 (10335, 18252) [45.5]	1939 (1448, 2430) [43.7]	**7.31** (6.07, 8.79)	10812 (6873, 14750) [59.5]	1751 (1189, 2313) [51.8]	**5.88** (4.37, 7.91)
**C**_**48**_ (fmol/punch)	14558 (11677, 17438) [35.0]	1936 (1488, 2384) [40.6]	**7.52** (6.76, 8.37)	7385 (4239, 10531) [69.8]	990.28 (518.10, 1462) [65.6]	**7.29** (4.45, 12.03)
**C**_**72**_ (fmol/punch)	14599 (11664, 17535) [35.4]	1780 (1370, 2191) [40.8]	**8.20** (7.39, 9.10)	5972 (3373, 8571) [70.2]	693.58 (319.46, 1068) [72.1]	**8.79** (4.54, 17.00)
**C**_**96**_ (fmol/punch)	14555 (11282, 17829) [40.0]	1725 (1210, 2240) [51.0]	**8.44** (7.17, 9.93)	4306 (1873, 6739) [85.2]	349.09 (179.55, 518.63) [58.8]	**10.45** (6.02, 18.15)
**AUC**_**last**_ (fmol.h/punch)	4275353 (3348643, 5202063) [38.0]	532210 (418319, 646101) [37.8]	**8.03** (7.47, 8.63)	1200458 (668398, 1732518) [72.6]	153337 (93968, 212706) [59.7]	**7.59** (4.89, 11.79)
**T**_**last**_ (h)	336 (336, 336) [0]	336 (336, 336) [0]	1.00 (1.00, 1.00)	319.77 (296.25, 343.29) (13.9)	211.92 (144.25, 279.58) [48.5]	1.50 (0.99, 2.27)
**t½** (h)	551.14 (134.09, 968.20) [92.3]	361.42 (−503.08, 1225.91) [177.0]	1.23 (0.51, 2.97)	70.81 (56.06, 85.55) (35.1)	52.36 (21.72, 83.01) [74.2]	1.13 (0.75, 1.71)

Geometric Mean Ratios (GMR) denote differences in pharmacokinetic parameters derived from paired HS-C and WM-DBS samples, with significant differences highlighted in bold text.

**Table 3:** Geometric mean (95% CI)[%CV] pharmacokinetic parameters for TFV_TAF_, FTC, DTG (Arm 1) and TFV_TDF_, 3TC, DTG (Arm 2) in HS-pL* and L-pL samples over 14 days drug intake cessation in healthy volunteers.

	Arm 1								
	TFV_TAF_			FTC			DTG		
	HS-pL	L-pL	GMR (95%CI)	HS-pL	L-pL	GMR (95% CI)	HS-pL	L-pL	GMR (95% CI)
**C**_**max**_ **(ng/mL)**	43.39 (31.34, 55.44) (48.5)	20.19 (14.71, 25.68) [49.0]	**2.15** (1.73, 2.67)	2887.87 (2388.66, 3387.09) [32.3]	1837.17 (1519.79, 2154.55) [32.3]	**1.57** (1.48, 1.67)	5129.83 (4149.84, 6109.82) [35.4]	3650.64 (2908.32, 4392.97) [37.7]	**1.41** (1.26, 1.57)
**T**_**max**_ **(h)**	1.32 (0.69, 1.95) (77.6)	1.45 (0.75, 2.14) [76.3]	0.91 (0.67, 1.23)	1.59 (0.85, 2.33) [73.2]	1.59 (0.85, 2.33) [73.2]	1.00 (1.00, 1.00)	2.09 (1.31, 2.88) [59.6]	2.52 (1.78, 3.26) [48.8]	0.83 (0.59, 1.17)
**C**_**24**_ **(ng/mL)**	12.39 (8.64, 16.14) (54.3)	8.90 (6.38, 11.42) [47.6]	**1.39** (1.08, 1.79)	102.09 (87.36, 116.83) [27.6]	75.68 (66.73, 84.62) [22.8]	**1.35** (1.23, 1.48)	1367.01 (650.62, 2083.39) [82.9]	1032.22 (670.05, 1394.39) [59.7]	**1.32** (1.20, 1.46)
**C**_**48**_ **(ng/mL)**	8.42 (6.66, 10.19) (38.3)	5.38 (3.39, 7.38) [56.3]	**1.53** (1.09, 2.14)	44.01 (37.22, 50.80) [29.3]	31.59 (26.34, 36.85) [30.3]	**1.37** (1.15, 1.63)	418.05 (171.16, 664.93) [87.4]	308.36 (131.99, 484.74) [86.2]	**1.36** (1.16, 1.59)
**C**_**72**_ **(ng/mL)**	6.87 (−1.70, 15.43) (157.2)	4.47 (3.13, 5.81) [50.6]	**1.67** (1.26, 2.21)	25.79 (3.62, 47.95) [124.8]	16.89 (14.02, 19.76) [31.9]	**1.53** (1.10, 2.12)	147.20 (−2.52, 296.91) [121.1]	95.30 (−1.18, 191.77) [124.3]	**1.54** (1.35, 1.76)
**C**_**96**_ **(ng/mL)**	4.81 (−0.27, 9.90) (140.5)	3.20 (2.17, 4.23) [51.3]	**1.58** (1.07, 2.35)	12.78 (10.56, 15.00) [32.7]	9.34 (7.73, 10.94) [32.5]	**1.37** (1.22, 1.53)	69.91 (−0.98, 140.81) [124.2]	46.03 (−6.15, 98.21) [140.5]	**1.52** (1.15, 2.00)
**AUC**_**last**_ **(ng.h/mL)**	1576.01 (754.54, 2397.47) (85.2)	889.28 (595.95, 1182.60) [53.7]	**1.77** (1.36, 2.31)	31393.54 (26856.21, 35930.87) [27.5]	20590.69 (17637.65, 23543.73) [27.3]	**1.52** (1.43, 1.62)	115066.88 (75916.03, 154217.74) [58.9]	82884.87 (58651.27, 107118.47) [51.8]	**1.39** (1.26, 1.53)
**T**_**last**_ **(h)**	292.50 (257.30, 327.71) (23.0)	234.24 (183.77, 284.71) [38.2]	1.25 (0.99, 1.57)	254.64 (211.53, 297.75) [31.7]	202.11 (163.19, 241.02) [36.1]	**1.26** **(1.07, 1.48)**	122.24 (71.03, 173.46) [69.5]	174.15 (140.52, 207.78) [36.1]	**0.70** (0.53, 0.92)
**t½** **(h)**	67.31 (32.18, 102.43) (80.8)	58.27 (35.72, 80.83) [62.6]	1.16 (0.74, 1.81)	49.22 (36.67, 61.78) [45.1]	43.93 (31.27, 56.58) [51.7]	1.12 (0.89, 1.41)	20.09 (9.90, 30.28) [80.1]	22.63 (16.49, 28.76) [48.8]	0.89 (0.65, 1.22)
	**Arm 2**								
	**TFV**_**TDF**_			**3TC**			**DTG**		
	**HS-pL**	**L-pL**	**GMR (95% CI)**	**HS-pL**	**L-pL**	**GMR (95% CI)**	**HS-pL**	**L-pL**	**GMR (95% CI)**
**C**_**max**_ **(ng/mL)**	389.06 (304.59, 473.52) [37.9]	287.65 (216.70, 358.59) [42.4]	**1.35** (1.18, 1.55)	2477.57 (1813.90, 3141.24) [45.3]	1495.65 (1155.18, 1836.11) [39.7]	**1.66** (1.53, 1.79)	4421.98 (3144.60, 5699.36) [49.0]	4254.76 (2825.93, 5683.59) [56.5]	1.04 (0.87, 1.24)
**T**_**max**_ **(h)**	1.64 (0.86, 2.42) [72.0]	1.81 (1.00, 2.62) [67.4]	0.91 (0.76, 1.08)	2.21 (1.40, 3.02) [56.8]	2.44 (1.66, 3.22) [50.9]	0.91 (0.76, 1.08)	3.28 (2.71, 3.85) [30.5]	3.73 (0.81, 6.65) [111.5]	0.88 (0.70, 1.11)
**C**_**24**_ **(ng/mL)**	70.48 (54.62, 86.35) [37.0]	67.02 (54.62, 79.41) [33.2]	1.08 (0.97, 1.21)	114.20 (60.51, 167.90) [65.8]	78.85 (54.96, 102.74) [49.8]	**1.42** (1.15, 1.75)	1423.72 (620.48, 2226.95) [82.4]	1296.61 (589.65, 2003.58) [80.6]	1.12 (0.88, 1.42)
**C**_**48**_ **(ng/mL)**	37.11 (27.07, 47.16) [45.2]	27.93 (21.48, 34.37) [40.8]	**1.31** (1.09, 1.57)	51.66 (34.58, 68.74) [52.8]	31.82 (24.48, 39.15) [39.3]	**1.62** (1.34, 1.96)	464.94 (210.71, 719.18) [80.2]	364.52 (180.80, 548.24) [74.7]	**1.31** (1.13, 1.52)
**C**_**72**_ **(ng/mL)**	16.55 (11.67, 21.44) [47.1]	12.52 (8.43, 16.61) [53.9]	**1.31** (1.14, 1.50)	29.44 (18.50, 40.39) [58.4]	15.83 (10.73, 20.93) [52.1]	1.86 (1.65, 2.10)	180.80 (96.92, 264.68) [73.3]	116.45 (58.87, 174.04) [72.4]	**1.55** (1.21, 1.99)
**C**_**96**_ **(ng/mL)**	7.11 (3.79, 10.42) [63.9]	5.54 (3.03, 8.05) [65.8]	1.30 (0.83, 2.02)	16.65 (11.19, 22.11) [52.5]	10.17 (8.14, 12.20) [34.8]	1.55 (1.19, 2.03)	53.57 (23.79, 83.34) [83.7]	38.45 (16.52, 60.38) [80.1]	**1.39** (1.10, 1.77)
**AUC**_**last**_ **(ng.h/mL)**	7398.03 (5827.44, 8968.62) [37.7]	5979.23 (4815.90, 7142.57) [34.8]	**1.24** (1.15, 1.33)	36347.37 (25794.30, 46900.45) [48.3]	21015.31 (16377.67, 25652.96) [38.6]	**1.72** (1.55, 1.93)	112226.76 (67325.16, 157128.36) [65.3]	97029.14 (59474.54, 134583.74) [62.7]	1.16 (0.97, 1.39)
**T**_**last**_ **(h)**	278.27 (234.60, 321.94) [28.3]	278.27 (234.60, 321.94) [28.3]	1.00 (0.77, 1.31)	264.83 (218.27, 311.40) [31.4]	183.46 (141.85, 225.06) [40.3]	**1.44** **(1.17, 1.78)**	134.72 (90.22, 179.22) [56.2]	169.61 (143.32, 195.90) [28.7]	0.78 (0.62, 1.01)
**t½** **(h)**	77.14 (−451.74, 606.01) [293.8]	45.24 (31.74, 58.74) [49.2]	1.72 (0.96, 3.08)	64.01 (44.13, 83.88) [50.8]	42.11 (26.15, 58.07) [63.0]	1.52 (1.01, 2.30)	21.29 (8.92, 33.65) [89.8]	19.91 (17.21, 22.61) [25.2]	1.07 (0.78, 1.46)

Significant differences in GMR are highlighted in bold.

### Nucleoside di-/triphosphate pharmacokinetics

Maximum TFV-DP concentrations (C_max_) in HS-C were ~12-fold higher (p < 0.001) in participants that received TDF (17166 fmol/sample) compared to those receiving TAF (1374 fmol/sample) and were quantifiable for a longer period post-cessation (336 versus. 259.2 hours). TFV-DP was undetectable in all WM-DBS from the TAF arm. TFV-DP C_max_ was ~ 7.6-fold higher in HS-C compared with WM-DBS (2271 fmol/sample) and quantifiable in all DBS up to 336 hours. The elimination of TFV-DP appeared to be prolonged in HS-C (t½ = 23 days) compared to WM-DBS (t½ = 15 days), but this was not significant [GMR = 1.23 (0.51, 2.97)].

FTC-TP C_max_ concentrations were ~4-fold higher in HS-C (7460 fmol/sample) compared with WM-DBS (1900 fmol/sample) and t½ was extended (3 days) in relation to WM-DBS (21 hours).

FTC-TP was quantifiable in HemaSep for up to 12 days post-cessation, compared to only 3.8 days in WM-DBS. For 3TC-TP, HS-C levels were ~7-fold higher and quantifiable for up to 13 days post-cessation, but the rate of elimination was comparable between the DBS sample types (HS-C ~ 3 days; WM-DBS ~ 2 days).

Nucleoside di-/triphosphate concentrations measured in WM-DBS and HS-C were significantly correlated: TFV-DP (r^2^ = 0.6609, P = < 0.0001), FTC-TP (r^2^ = 0.4281, P = < 0.0001), 3TC-TP (r^2^ = 0.6890, P = < 0.0001) (S1 Fig). For TFV-DP, correlation analysis could only be performed in the TDF arm as all WM-DBS samples were <LLQ in the TAF arm.

### NRTI pharmacokinetics

TFV C_max_ HS-pL and L-pL concentrations were 9-fold and 14-fold higher in participants receiving TDF versus TAF. When comparing the sampling methods, HS-pL exposures (AUC_last_) were 1.77 (TFV_TAF_) and 1.24-fold (TFV_TDF_) higher than corresponding L-pL exposures, over the course of the sampling period (Table 3).

For FTC, HS-pL exposures were approximately 1.5-fold higher than L-pL. The FTC t½ was comparable for HS-pL and L-pL (2.1 days, 1.8 days). 3TC exposures in HS-pL were ~1.7-fold greater than L-pL. Interestingly, HS-pL exhibited a longer window of drug detection, with a T_last_ of 11 days, compared to L-pL (7.7 days) post-final dose, as well as a prolonged t_1/2_ (2.7 days, 1.8 days).

NRTI measurements in HS-pL and L-pL were significantly correlated: TFV_TAF_ (r^2^ = 0.6528, P = < 0.0001), TFV_TDF_ (r^2^ = 0.9623, P = < 0.0001), FTC (r^2^ = 0.9566, P = < 0.0001) and 3TC (r^2^ = 0.9578, P = < 0.0001) (S2 Fig A-D).

### DTG pharmacokinetics

DTG (50 mg) was measured in both arms. HS-pL displayed a 1.4-fold increase in DTG exposure compared to L-pL in those receiving TAF, but no differences were noted in the TDF arm. DTG t_1/2_ (~20 hours) was comparable in both arms and matrices (Table 3). In relation to known therapeutic targets for DTG, HS-pL (and L-pL) concentrations remained above the recommended minimum effective concentration (MEC) (324 ng/mL) [15] in 69% (and 55%) of participants at 24 hours post-cessation (equivalent to C_48_ timepoint). However, at 48- and 72-hours post-cessation (equivalent to C_72_ and C_96_ timepoints) only 17% (and 7%) and 3% (and 3%) participants were above the MEC. DTG measurements in HS-pL and L-pL were significantly correlated (r^2^ = 0.9544, P = < 0.0001) (S2E Fig).

### Bland Altman plots

Bland Altman plots for nucleoside di-/triphosphates, with HS-C plotted against WM-DBS, demonstrated a consistent positive bias with HS-C yielding higher concentrations than WM-DBS for all three analytes. The mean difference was greatest for TFV-DP (7.67), followed by 3TC-TP (6.20) and FTC-TP (2.82) as shown in Fig 4A–C. Although most measurements fell within the limits of agreement there was evidence of “concentration dependency”, with most outliers located at higher concentrations.

Bland Altman analysis for the NRTI and DTG (Fig 5A-E), with HS-pL plotted against L-pL, showed good agreement between HS-pL and liquid plasma for all parent drugs, with a small positive bias (mean difference 1.23–1.65). Most observations fell within the limits of agreement, with no evidence of meaningful proportional bias.

## Discussion

The APT-POCT-01 trial evaluated NRTI and DTG PK in whole blood, plasma, urine, and saliva [7]. This sub-study focused on the kinetics of NRTI phosphorylated metabolites in paired WM-DBS and HS-DBS.

Reported t_1/2_ for NRTI phosphorylated metabolites in this study align with prior estimates in WM-DBS, including approximately 17 days for TFV-DP and 1.5 days for FTC-TP. Notably, this study is the first to characterize 3TC-TP kinetics in DBS, with a t_1/2_ of 3 days in HS-C and 2 days in WM-DBS. These data can be used to support clinicians in making adherence assessments for PLWH who are taking 3TC-based regimens, such as TFV/3TC/DTG (TLD), which is one of the first-line WHO recommended treatments for HIV [16]. The rapid elimination of 3TC-TP and FTC-TP makes them suitable for short-term adherence monitoring [17], whilst prolonged elimination of TFV-DP reinforces its utility for assessing longer-term adherence [18,19]. The longer apparent half-lives observed in HS-C compared with WM-DBS could be seen as an analytical effect, whereby HS-C higher concentrations allow quantification further into the terminal elimination phase, whilst lower WM-DBS concentrations truncate this phase, leading to a shorter estimated half-life [17].

The marked difference in TFV-DP concentrations observed between participants receiving TDF and TAF, reflected in a 12-fold higher C_max_ of TFV-DP in HS-C in the TDF arm, underscores the impact of formulation on intracellular TFV-DP accumulation. This was reinforced by our inability to detect TFV-DP levels in all WM-DBS samples from the TAF arm. These results are consistent with our previous data showing ~15-fold higher TFV-DP levels in HS-DBS from TDF- versus TAF-based ART [10] and with data from another WM-DBS study, where pooling of WM-DBS sub-punches was required to increase assay sensitivity and enable quantification of TFV-DP in individuals receiving TAF [20]. A potential explanation is due to differences in enzymatic activity for each analyte. Lysosomal carboxypeptidase A (cathepsin A) is an enzyme localised within peripheral blood mononuclear cells (PBMC) which has been recognised as the primary hydrolase responsible for activation of TAF intracellularly. TAF requires hydrolysis by cathepsin A into its parent moiety, which is then phosphorylated into its active form (TFV-DP) by intracellular kinases, which leads to an accumulation of TFV-DP within PBMC [21]. However, cathepsin A is not present in RBC, therefore hydrolysis does not take place in these cells, resulting in reduced concentrations of TFV and therefore, TFV-DP. Conversely, the metabolism of TDF is not dependent on cathepsin A activity, as it is phosphorylated intracellularly by nucleoside diphosphate kinase and AMP kinase into TFV-DP [22], leading to increased TFV-DP concentrations in RBC.

The comparison between WM-DBS and HS-DBS (TDF arm) revealed approximately 7-fold higher TFV-DP levels in HS-C, with the HS-DBS method yielding systematically higher values, particularly at the upper concentration ranges. This discrepancy likely stems from differences in card materials, applied blood volumes (100 µL for HemaSep vs. 50 µL for Whatman) and thus higher cell densities per unit area, and sub-punch sizes (12 mm for HemaSep vs. 6 mm for Whatman). Despite these variations, positive and significant correlations were observed between the two methods, and most measurements were within the limits of agreement via Bland-Altman analysis, indicating good overall concordance. However, it is important to recognise that Bland–Altman are typically used for cross-validation between methods or laboratories measuring the same “true” sample. In this context, HS-C and WM-DBS represent distinct specimen types rather than interchangeable measures of a single reference matrix, and therefore identical absolute concentrations are not expected. Accordingly, the Bland–Altman analysis in this study should be interpreted as assessing agreement and systematic differences between the sampling approaches, rather than as a validation of absolute accuracy against a gold standard.

The observed differences between traditional DBS and plasma separation cards, as well as the distinct PK profiles between TDF and TAF, have important implications for interpreting adherence from intracellular TFV-DP concentrations. Lower TFV-DP DBS concentrations in those receiving TAF may lead to underestimation of adherence if the sampling approach and regimen aren’t considered. This reinforces the need for developing drug regimen and matrix-specific adherence thresholds to ensure accurate clinical interpretation and avoid misclassification of adherence status. HemaSep could serve as a useful tool for adherence monitoring for those receiving TAF-based regimens and in individuals receiving on-demand PrEP where TFV-DP concentrations are expected to be lower.

Previous studies identifying adherence benchmarks for TFV-DP and FTC-TP have been performed using WM-DBS [1,2,23]. Pharmacokinetic analyses from the iPrEx study, for example, identified TFV-DP concentrations in WM-DBS of: ~ 350 fmol/punch for 2 doses/week, ~ 700 fmol/punch for 4 doses/week, and ≥1250 fmol/punch for daily dosing (6–7 doses/week). Based on our paired data, equivalent adherence thresholds in the cellular fraction of HS-DBS would be approximately 5-fold (FTC-TP) to 8-fold (TFV-DP_TDF_) higher than those derived from WM-DBS, though these differences are influenced by the volume of blood applied and the punch size used for extraction.

HS-DBS are advantageous in that they enable the simultaneous quantification of parent NRTI and their nucleoside di-/triphosphates from one sample as a measure of both short- and long-term adherence. The novel plasma separation technology also lends itself to applications for ‘at-home’ testing, which allows for patient-centric sampling to be performed outside clinical settings via finger prick kits. In contrast, WM-DBS drug concentrations are influenced by drug-specific red blood cell partitioning, variations in haematocrit and dilution effects from the cellular components of whole blood, such that correction factors need to be applied to align with relatable liquid plasma values.

NRTI concentrations in HS-pL reported in this study align with L-pL levels previously reported [24–27]. Similarly, higher TFV concentrations in the TDF arm were expected due to the higher TDF dose (300 mg TDF vs. 25 mg TAF) and are consistent with previous findings [28]. The quantification of the TAF pro-drug itself was not included in the PK analysis due to most samples reading <LLQ for both HS-pL and L-pL. This was expected due to rapid elimination of TAF from plasma (t_1/2_ ~ 25 minutes) [29]. The half-lives of the parent NRTI drugs observed in this study were longer than previously reported (14 hr for TFV, 10 hr for FTC, 3–4 hr for 3TC) [1,15,27], likely because the half-lives were determined over a 14-day period, which includes the terminal elimination phase as concentrations approach the assay LLQ.

NRTI exposures in HS-pL were between 1.2 to 1.8-fold higher than in L-pL across the sampling period, with TFV levels in HS-pL up to 2-fold higher during the C_max_ phase (TAF arm). Although, drug absorption and elimination kinetics (T_max_, t½) in HS-pL were largely consistent with paired L-pL data. Significant positive correlations were observed between HS-pL and L-pL concentrations for all NRTI (P < 0.0001). Due to their relatively short elimination half-lives, direct quantification of NRTI parent compounds in plasma are a reliable indicator of recent dosing, and HS-pL may serve as a viable alternative matrix for this purpose in remote settings where collection, transport and processing of liquid plasma is not practicable.

The strong correlation between HS-pL and L-pL DTG concentrations (r² = 0.9544, P < 0.0001) supports the suitability of HS-DBS for short-term PK monitoring. DTG exposures were ~1.4 fold higher in HS-pL compared to L-pL in the TAF arm, though both arms and sampling types demonstrated similar elimination kinetics (DTG t_1/2_ ~ 20 hours). Therapeutic levels were maintained, with comparable proportions of participants above the target DTG concentration (MEC: 324 ng/mL) in both matrices. However, as the MEC is defined using liquid plasma, comparisons of proportions above this threshold in HS-pL are intended to be descriptive and to contextualise relative trends between matrices, rather than to imply direct clinical equivalence. Inter-subject variability, as well as systematically higher levels of parent drug in HS-pL compared with liquid plasma, as reflected by higher GMRs, likely stems from methodological factors, including excision and extraction of the entire plasma ring and uncertainty in the “partitioned” plasma volume which is potentially influenced by haematocrit and blood viscosity.

Further studies are warranted to establish adherence thresholds for HS-DBS using simulated dosing regimens, or through validation in real-world patient cohorts, in both treatment and PrEP contexts. For DTG there is particular interest in understanding whether its intracellular half-life is prolonged relative to the parent drug in plasma – which our data indicate, falls below target after 48 hours. In this context, HS-DBS could offer a practical alternative to PBMC isolation for assessing intracellular DTG levels (in the central spot), while simultaneously enabling measurement of plasma concentrations from the same card (via the outer ring). HS-DBS offer a logistically simpler, more scalable approach that could feasibly be implemented in remote settings to support such strategies.

## Conclusion

In conclusion, this study offers important insights into the pharmacokinetics of NRTIs and their active phosphorylated metabolites across different blood matrices following treatment cessation, aiding the validation of adherence benchmarks. Plasma separation cards, which allow simultaneous quantification of parent drugs (for short-term adherence) and nucleoside triphosphates (for long-term adherence) from both plasma and cellular fractions, represent a practical alternative to conventional sampling methods like liquid plasma and whole blood DBS. The observed differences in TFV-DP levels between TDF and TAF regimens, and between DBS sampling approaches, highlight the importance of regimen and matrix-specific interpretation in DBS adherence monitoring.

## Supporting information

S1 Fig Correlation of quantifiable nucleoside di-/triphosphate concentrations in HS-C and WM (expressed in fmol/punch) for (A) FTC-TP, (B) TFV-DP_TDF_, and (C) 3TC-TP. Data are log transformed. All correlations were significant (p = < 0.001).
(TIF)

S2 Fig Correlation of quantifiable dried plasma (HS-pL) and liquid plasma concentrations (expressed in ng/mL) for (A) TFV_TAF_, (B) FTC, (C) TFV_TDF_, (D) 3TC and (E) DTG. Data are log transformed. All correlations were significant (p = < 0.001).
(TIF)

S3 Fig Individual participant profiles displaying concentrations of nucleoside di-/triphosphates over the study washout period (0–336 hours post-cessation). Comparison of arms 1 and 2 (TFV_TAF_, TFV-DP_TDF_) (A) indicates greater concentrations of TFV-DP in arm 2. Matrix-specific comparisons are found graphs B (TFV-DP), C (FTC-TP) and D (3TC-TP).
(TIF)

S4 Fig Individual participant profiles displaying concentrations of TFVTAF (A), TFV-DPTDF (B), FTC (C), 3TC (D) and DTG (E – arm 1, F – arm 2). Comparison of paired HS-pL and L-pL samples over the study washout period (0 – 336 hours post-cessation).
(TIF)

## References

[1] Anderson PL, Liu AY, Castillo-Mancilla JR, Gardner EM, Seifert SM, McHugh C, et al. Intracellular Tenofovir-Diphosphate and Emtricitabine-Triphosphate in Dried Blood Spots following Directly Observed Therapy. Antimicrob Agents Chemother. 2017;62(1):e01710-17. doi: 10.1128/AAC.01710-17 29038282 PMC5740314

[2] Castillo-Mancilla J, Seifert S, Campbell K, Coleman S, McAllister K, Zheng J-H, et al. Emtricitabine-Triphosphate in Dried Blood Spots as a Marker of Recent Dosing. Antimicrob Agents Chemother. 2016;60(11):6692–7. doi: 10.1128/AAC.01017-16 27572401 PMC5075074

[3] Grant RM, Lama JR, Anderson PL, McMahan V, Liu AY, Vargas L, et al. Preexposure chemoprophylaxis for HIV prevention in men who have sex with men. N Engl J Med. 2010;363(27):2587–99. doi: 10.1056/NEJMoa1011205 21091279 PMC3079639

[4] Arrington-Sanders R, Wilson CM, Perumean-Chaney SE, Patki A, Hosek S. Brief Report: Role of Sociobehavioral Factors in Subprotective TFV-DP Levels Among YMSM Enrolled in 2 PrEP Trials. J Acquir Immune Defic Syndr. 2019;80(2):160–5. doi: 10.1097/QAI.0000000000001901 30640203 PMC6486465

[5] Hosek SG, Rudy B, Landovitz R, Kapogiannis B, Siberry G, Rutledge B, et al. An HIV Preexposure Prophylaxis Demonstration Project and Safety Study for Young MSM. J Acquir Immune Defic Syndr. 2017;74(1):21–9. doi: 10.1097/QAI.0000000000001179 27632233 PMC5140725

[6] Cottrell ML, Yang KH, Prince HMA, Sykes C, White N, Malone S, et al. A Translational Pharmacology Approach to Predicting Outcomes of Preexposure Prophylaxis Against HIV in Men and Women Using Tenofovir Disoproxil Fumarate With or Without Emtricitabine. J Infect Dis. 2016;214(1):55–64. doi: 10.1093/infdis/jiw077 26917574 PMC4907409

[7] Else LJ, Dickinson L, Edick S, Zyhowski A, Ho K, Meyn L, et al. Tenofovir, emtricitabine, lamivudine and dolutegravir concentrations in plasma and urine following drug intake cessation in a randomized controlled directly observed pharmacokinetic trial to aid point-of-care testing. J Antimicrob Chemother. 2024;79(7):1597–605. doi: 10.1093/jac/dkae147 38758205 PMC11215529

[8] Hopewell S, Chan A-W, Collins GS, Hróbjartsson A, Moher D, Schulz KF, et al. CONSORT 2025 statement: updated guideline for reporting randomised trials. BMJ. 2025;389:e081123. doi: 10.1136/bmj-2024-081123 40228833 PMC11995449

[9] Bioanalytical method validation and study sample analysis - guidance for industry. 2022.

[10] Thompson B, Dilly-Penchala S, Amara A, Reynolds H, Khoo S, Else L. Application of novel plasma separation filter cards for quantification of nucleoside/nucleotide reverse transcriptase inhibitor di/triphosphates in dried blood spots using LC-MS. Bioanalysis. 2023;15(13):739–56. 37293769 10.4155/bio-2023-0057 PMC10463213

[11] Waitt C, Diliiy Penchala S, Olagunju A, Amara A, Else L, Lamorde M, et al. Development, validation and clinical application of a method for the simultaneous quantification of lamivudine, emtricitabine and tenofovir in dried blood and dried breast milk spots using LC-MS/MS. J Chromatogr B Analyt Technol Biomed Life Sci. 2017;1060:300–7. doi: 10.1016/j.jchromb.2017.06.012 28651173 PMC5588985

[12] Jackson A, Moyle G, Watson V, Tjia J, Ammara A, Back D, et al. Tenofovir, emtricitabine intracellular and plasma, and efavirenz plasma concentration decay following drug intake cessation: implications for HIV treatment and prevention. J Acquir Immune Defic Syndr. 2013;62(3):275–81. doi: 10.1097/QAI.0b013e3182829bd0 23274933

[13] Penchala SD, Fawcett S, Else L, Egan D, Amara A, Elliot E, et al. The development and application of a novel LC-MS/MS method for the measurement of Dolutegravir, Elvitegravir and Cobicistat in human plasma. J Chromatogr B Analyt Technol Biomed Life Sci. 2016;1027:174–80. doi: 10.1016/j.jchromb.2016.05.040 27290668

[14] Billett HH. Hemoglobin and hematocrit. Clinical Methods: The History, Physical, and Laboratory Examinations. 3rd ed. Boston. 1990. 21250045

[15] Cottrell ML, Hadzic T, Kashuba ADM. Clinical pharmacokinetic, pharmacodynamic and drug-interaction profile of the integrase inhibitor dolutegravir. Clin Pharmacokinet. 2013;52(11):981–94. doi: 10.1007/s40262-013-0093-2 23824675 PMC3805712

[16] Tegegne BA, Alehegn AA, Kassahun M. Drug Use Evaluation of Tenofovir/Lamivudine/Dolutegravir (TLD) Fixed-Dose Combination for Initiation and Transition Among HIV-Infected Individuals Attending Lumame Primary Hospital, North West Ethiopia. Integr Pharm Res Pract. 2024;13:31–42. doi: 10.2147/IPRP.S455351 38650710 PMC11034558

[17] Thompson B. Development and application of novel dried blood spot methods for the quantification of antiretroviral drugs and their metabolites: University of Liverpool; 2024.

[18] Zheng J-H, Guida LA, Rower C, Castillo-Mancilla J, Meditz A, Klein B, et al. Quantitation of tenofovir and emtricitabine in dried blood spots (DBS) with LC-MS/MS. J Pharm Biomed Anal. 2014;88:144–51. doi: 10.1016/j.jpba.2013.08.033 24055850 PMC3842403

[19] Schauer AP, Sykes C, Cottrell ML, Prince H, Kashuba ADM. Validation of an LC-MS/MS assay to simultaneously monitor the intracellular active metabolites of tenofovir, emtricitabine, and lamivudine in dried blood spots. J Pharm Biomed Anal. 2018;149:40–5. doi: 10.1016/j.jpba.2017.10.030 29100029 PMC5741486

[20] Yager J, Castillo-Mancilla J, Ibrahim ME, Brooks KM, McHugh C, Morrow M, et al. Intracellular Tenofovir-Diphosphate and Emtricitabine-Triphosphate in Dried Blood Spots Following Tenofovir Alafenamide: The TAF-DBS Study. J Acquir Immune Defic Syndr. 2020;84(3):323–30. doi: 10.1097/QAI.0000000000002354 32539288

[21] Birkus G, Wang R, Liu X, Kutty N, MacArthur H, Cihlar T. Cathepsin A is the major hydrolase catalyzing the intracellular hydrolysis of the antiretroviral nucleotide phosphonoamidate prodrugs GS-7340 and GS-9131. Antimicrob Agents Chemother. 2007;51(2):543–50. 17145787 10.1128/AAC.00968-06 PMC1797775

[22] Robbins BL, Greenhaw J, Connelly MC, Fridland A. Metabolic pathways for activation of the antiviral agent 9-(2-phosphonylmethoxyethyl)adenine in human lymphoid cells. Antimicrob Agents Chemother. 1995;39(10):2304–8. doi: 10.1128/AAC.39.10.2304 8619586 PMC162933

[23] Castillo-Mancilla JR, Zheng J-H, Rower JE, Meditz A, Gardner EM, Predhomme J, et al. Tenofovir, emtricitabine, and tenofovir diphosphate in dried blood spots for determining recent and cumulative drug exposure. AIDS Res Hum Retroviruses. 2013;29(2):384–90. doi: 10.1089/AID.2012.0089 22935078 PMC3552442

[24] Cressey TR, Siriprakaisil O, Kubiak RW, Klinbuayaem V, Sukrakanchana P-O, Quame-Amaglo J, et al. Plasma pharmacokinetics and urinary excretion of tenofovir following cessation in adults with controlled levels of adherence to tenofovir disoproxil fumarate. Int J Infect Dis. 2020;97:365–70. doi: 10.1016/j.ijid.2020.06.037 32553717 PMC7392195

[25] Podany AT, Bares SH, Havens J, Dyavar SR, O’Neill J, Lee S, et al. Plasma and intracellular pharmacokinetics of tenofovir in patients switched from tenofovir disoproxil fumarate to tenofovir alafenamide. AIDS. 2018;32(6):761–5. doi: 10.1097/QAD.0000000000001744 29334548 PMC5854526

[26] Adams JL, Sykes C, Menezes P, Prince HMA, Patterson KB, Fransen K, et al. Tenofovir diphosphate and emtricitabine triphosphate concentrations in blood cells compared with isolated peripheral blood mononuclear cells: a new measure of antiretroviral adherence?. J Acquir Immune Defic Syndr. 2013;62(3):260–6. doi: 10.1097/QAI.0b013e3182794723 23111578 PMC4042836

[27] Minzi O, Mugoyela V, Gustafsson L. Correlation between lamivudine plasma concentrations and patient self-reported adherence to antiretroviral treatment in experienced HIV patients. Ther Clin Risk Manag. 2011;7:441–6. doi: 10.2147/TCRM.S23625 22162920 PMC3233527

[28] Ray AS, Fordyce MW, Hitchcock MJM. Tenofovir alafenamide: A novel prodrug of tenofovir for the treatment of Human Immunodeficiency Virus. Antiviral Res. 2016;125:63–70. doi: 10.1016/j.antiviral.2015.11.009 26640223

[29] Kawuma AN, Wasmann RE, Sinxadi P, Sokhela SM, Chandiwana N, Venter WDF, et al. Population pharmacokinetics of tenofovir given as either tenofovir disoproxil fumarate or tenofovir alafenamide in an African population. CPT Pharmacometrics Syst Pharmacol. 2023;12(6):821–30. doi: 10.1002/psp4.12955 37013631 PMC10272303

