# Supplementary figures and images for: Pharmacokinetics of Dolutegravir, nucleoside analogues, and intracellular metabolites using plasma separation cards: A comparative analysis with traditional sampling methods in healthy volunteers
Source: PLoS One. 2026 Jan 23;21(1):e0341252. doi: 10.1371/journal.pone.0341252 (PMC12829806; doi:10.1371/journal.pone.0341252)

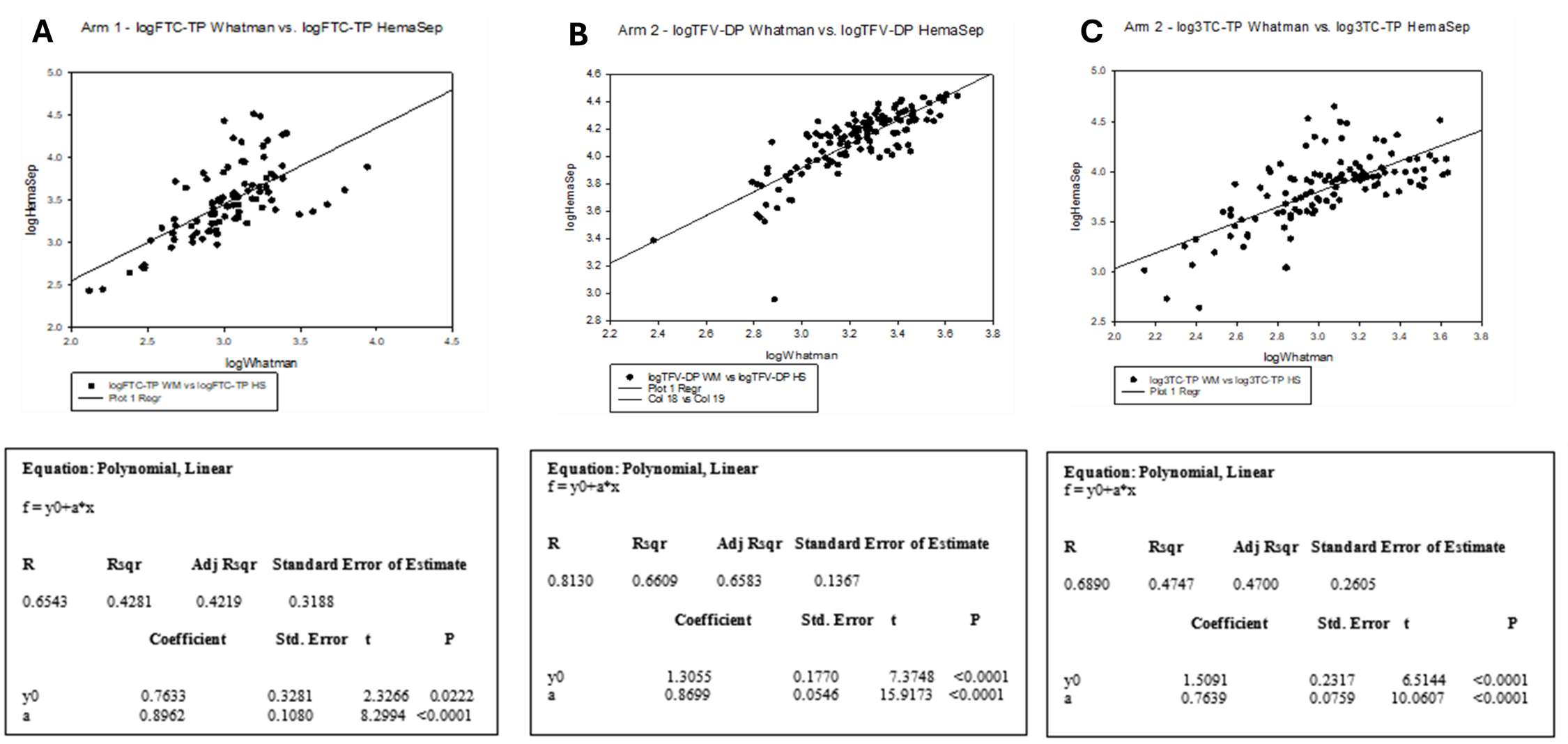

Supplement: S1 Fig — Data are log transformed. All correlations were significant (p = < 0.001). (TIF) [file pone.0341252.s001.tif]

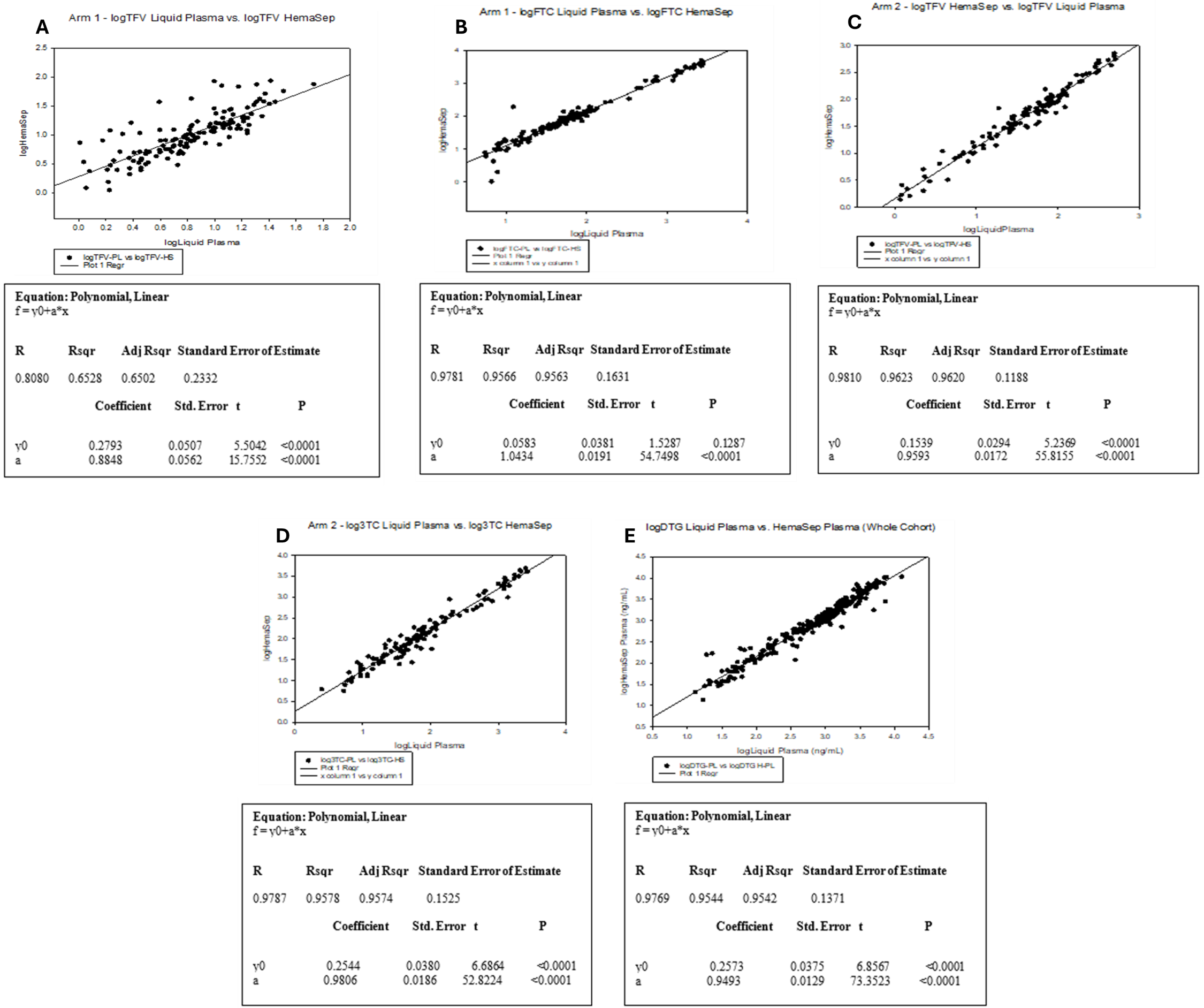

Supplement: S2 Fig — Data are log transformed. All correlations were significant (p = < 0.001). (TIF) [file pone.0341252.s002.tif]

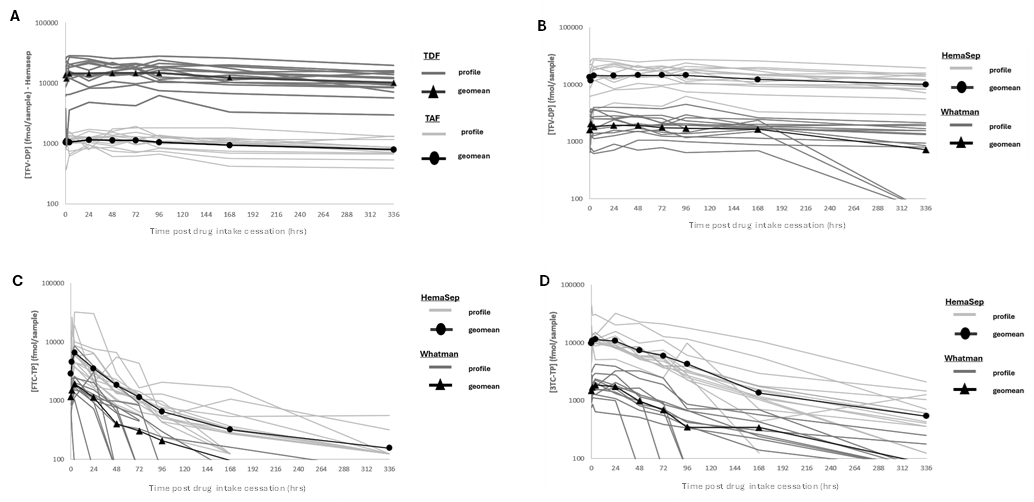

Supplement: S3 Fig — Comparison of arms 1 and 2 (TFVTAF, TFV-DPTDF) (A) indicates greater concentrations of TFV-DP in arm 2. Matrix-specific comparisons are found graphs B (TFV-DP), C (FTC-TP) and D (3TC-TP). (TIF) [file pone.0341252.s003.tif]

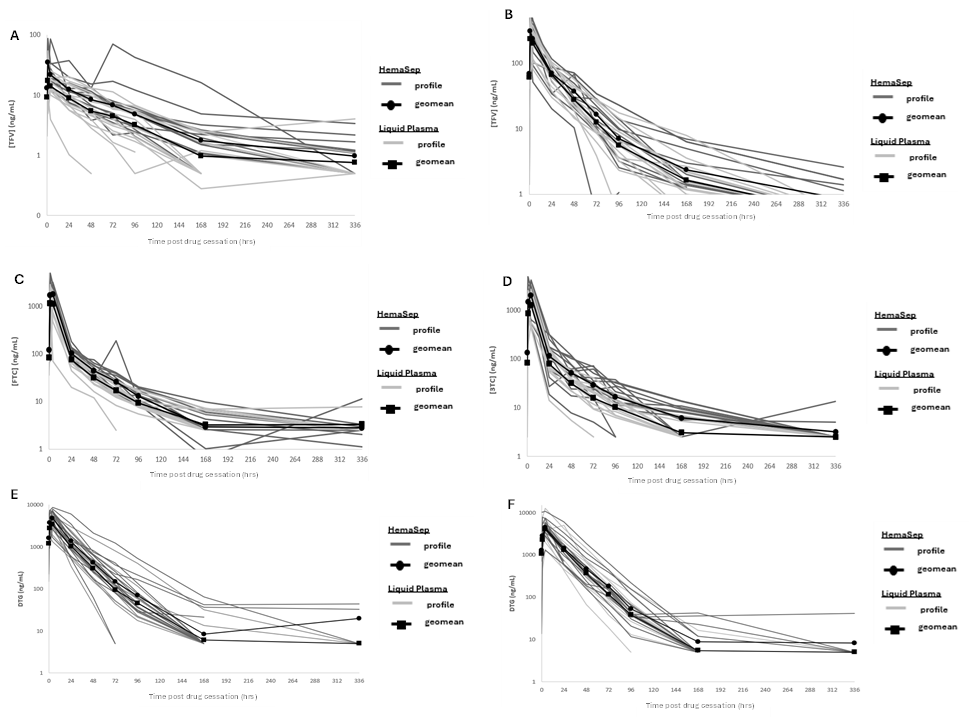

Supplement: S4 Fig — Comparison of paired HS-pL and L-pL samples over the study washout period (0 – 336 hours post-cessation). (TIF) [file pone.0341252.s004.tif]
